# Enhancing Cervical Cancer Screening: New Diagnostic Methodologies, Triage, and Risk Stratification in Prevention and Treatment

**DOI:** 10.3390/life15030367

**Published:** 2025-02-26

**Authors:** Nazira Kamzayeva, Gauri Bapayeva, Milan Terzic, Berik Primbetov, Balkenzhe Imankulova, Yevgeniy Kim, Arailym Sultanova, Kuralay Kongrtay, Nazira Kadroldinova, Talshyn Ukybassova

**Affiliations:** 1 Clinical Academic Department of Women’s Health, CF “University Medical Center”, Astana 010000, Kazakhstan; gauri.bapayeva@gmail.com (G.B.); milan.terzic@nu.edu.kz (M.T.); berik.p@gmail.com (B.P.); balkenzhe.imankulova2@umc.org.kz (B.I.); evg.kim94@gmail.com (Y.K.); kkongrtay@nu.edu.kz (K.K.); nazira.kadroldinova@nu.edu.kz (N.K.); talshynu@yandex.ru (T.U.); 2 Department of Surgery, School of Medicine, Nazarbayev University, Astana 010000, Kazakhstan; 3 Clinical Academic Department of Internal Medicine, University Health Center, CF “University Medical Center”, Astana 010000, Kazakhstan; sultanova001@list.ru

**Keywords:** human papillomavirus, HPV, cervical cancer, precancerous cervical lesions, screening, microbiota, self-sampling, diagnostic biomarkers, persistent HPV infection

## Abstract

Human papillomavirus (HPV) is a well-established etiological factor in the development of precancerous cervical lesions and cervical cancer. This narrative review synthesizes current evidence on the global prevalence, genotype distribution, and pathophysiological mechanisms of HPV infection, emphasizing regional epidemiological variations that influence prevention and treatment strategies. Particular attention is given to high-risk HPV genotypes, their role in carcinogenesis, and the impact of co-infections and the cervicovaginal microbiota on infection persistence and disease progression. Advances in diagnostic methodologies, including E6/E7 oncoprotein detection, DNA methylation, and microRNA-based assays, are examined in the context of improving screening accuracy and early detection. Furthermore, the review explores the psychological implications of HPV diagnosis and underscores the importance of integrating psychosocial support into clinical management. Given the challenges associated with screening coverage, the potential of self-sampling techniques, particularly in resource-limited settings, is discussed as a means to enhance accessibility and participation in cervical cancer prevention programs. By providing a comprehensive overview of these interrelated factors, this review highlights the necessity of a multidisciplinary approach that integrates novel diagnostic strategies, targeted prevention efforts, and supportive care to mitigate the burden of HPV-associated diseases.

## 1. Introduction

The human papillomavirus (HPV) is a double-stranded DNA virus belonging to the Papillomaviridae family and is specific to humans. It comprises a family of more than 200 subtypes with specific tissue tropism (either cutaneous or mucosal) [1]. HPV is classified into two categories based on its oncogenic potential: high-risk types, which cause precancerous lesions or cancer in the anogenital region (16, 18, 31, 33, 35, 39, 45, 51, 52, 56, 58, 59, 68, 73, and 82), and low-risk types, which lead to skin papillomas and genital warts (6, 11, 40, 42, 43, 44, 54, 61, 62, 71, 72, 81, 83, 84, 85, and 89) [1].

The association between HPV infection and malignancies is well established, particularly in relation to carcinomas of the anogenital region, including cervical cancer, vulvar cancer, colorectal cancer, as well as head and neck cancers, specifically oropharyngeal carcinoma [2,3,4,5,6,7].

HPV is a prevalent sexually transmitted infection worldwide. The global prevalence of HPV infections varies significantly based on factors such as age, gender, sexual activity, screening participation, the HPV test used, the number of genotypes detected, and the sampling site [1,2,3]. Approximately 12% of women globally have detectable cervical HPV infections, with prevalence rates varying by geography and age. In men, the global prevalence rate of genital HPV infection ranges from 3.5% to 45%, depending on the region and population studied. HPV infections are more common in younger individuals, with prevalence peaking between the ages of 20 and 24. The virus is highly prevalent in sub-Saharan Africa, where the prevalence among women is 24%, and in Eastern Europe, where it is 21% [2]. In the United States, about 26.8% of women aged 14 to 59 are infected with at least one type of HPV, with 15.2% infected with one or more high-risk types that can cause cancer. Globally, it is estimated that around 291 million women carry HPV DNA, with 32% infected with HPV16 or HPV18, or both [2,3,4,5,6,7,8].

Some researchers state that multiple infections significantly increase the risk of cervical cancer: alpha-9 genotypes elevate the risk by 5.3 times, while alpha-7 genotypes increase it by 2.5 times [8]. The other studies report that the risk of cervical intraepithelial neoplasia 2/3 (CIN2+) from multiple HPV infections is not higher than that associated with a single high-risk HPV infection, such as HPV 16. Some studies even indicate that multiple infections may be linked to a lower risk of CIN2+ compared to a solitary HPV16 infection [9,10,11]. Additionally, while multiple high-risk HPV infections have been associated with an increased likelihood of CIN1 and CIN2, they do not necessarily lead to a higher risk of CIN3. One study also found that multiple high-risk infections could raise the chances of high-grade squamous intraepithelial lesion (HSIL) persistence or recurrence after treatment compared to single infections. However, the overall evidence does not indicate a significantly greater carcinogenic risk from multiple HPV infections compared to a single high-risk type like HPV 16 [9,10,11]. These statistics highlight the widespread nature of HPV infections and underscore the importance of preventive measures such as vaccination and regular screening [11,12,13,14,15,16,17].

Worldwide, the seven most common HPV types associated with cervical cancer are 16, 18, 45, 33, 31, 52, and 58—all of which are covered by the nonavalent HPV vaccine, Gardasil 9 [18,19]. A study from South Africa supports this, identifying HPV types 16, 18, 45, 35, 33, 52, 31, and 58 as the most common in cervical cancer cases. These findings highlight the importance of understanding regional variations in HPV type distribution and ensuring vaccine strategies align with these patterns [19]. The most common combinations include HPV 16 and other oncogenic genotypes. Multiple infections demonstrate a synergistic effect, amplifying the risk of disease progression. The primary oncogenic genotypes globally are HPV 16, 18, 52, and 58. In Europe and the Americas, HPV 31 and 51 are also prevalent, while HPV 33 is common in Asia, and HPV 35 and 45 dominate in Africa. In Africa, HPV 35 is the fourth most prevalent HPV type in cervical cancer, detected in 10.8% (18/167) of cases, following HPV types 16, 18, and 45. This highlights the potential benefits of incorporating HPV 35 into future vaccine formulations to improve protection, particularly in areas where this genotype is common [8].

Recent studies show that conization significantly lowers the risk of cervical cancer, reducing it from 30% in untreated women to less than 1% in those who undergo treatment. However, persistent HPV infection after conization is associated with an 8% risk of recurrent high-grade disease within five years. HPV testing as a “test of cure” plays a critical role in risk assessment, as a positive result post-treatment indicates an increased likelihood of recurrence. Additionally, combining HPV testing with surgical margin evaluation enhances recurrence prediction, though it may be less specific than HPV testing alone. Long-term monitoring through HPV testing or co-testing every three years is recommended for at least 25 years post-treatment, emphasizing its importance in managing long-term risk [9,20,21].

Multiple high-risk HPV infections are more frequently observed in young women, individuals with immunodeficiency, or those with an early onset of sexual activity. These infections are associated with increased viral load and persistence, which contribute to lesion progression. Such infections are detected in 25% of women after treatment for HSIL, with their frequency influenced by diagnostic methods and epidemiological factors [11].

Before the introduction of HPV vaccines, HPV-related diseases were largely considered unpreventable [3]. The identification of HPV’s role in various benign and malignant conditions, along with the development of preventive vaccines, has significantly transformed disease prevention strategies. Prophylactic HPV vaccines effectively prevent HPV infections and related neoplastic conditions but do not offer therapeutic benefits or eliminate existing infections [2,3]. As of 2018, global HPV vaccination coverage was estimated at 12.2% [3]. Despite these efforts, millions of girls—particularly in low- and middle-income countries (LMICs)—remain unvaccinated. In 2018, cervical cancer claimed the lives of over 300,000 women, with 90% of these deaths occurring in LMICs. Among the 61 million girls who turned 15 that year, an estimated 7000 may develop and succumb to cervical cancer over their lifetime.

Despite multiple studies done on the area of HPV infection investigation and considerable achievement in this field, the topic remains relevant and up to date for discussion. Thus, in this review aims to summarize existing evidence on the impact of HPV on the risk of development and persistence of cervical lesions.

## 2. Methods

### 2.1. Literature Search

**Figure 1:**
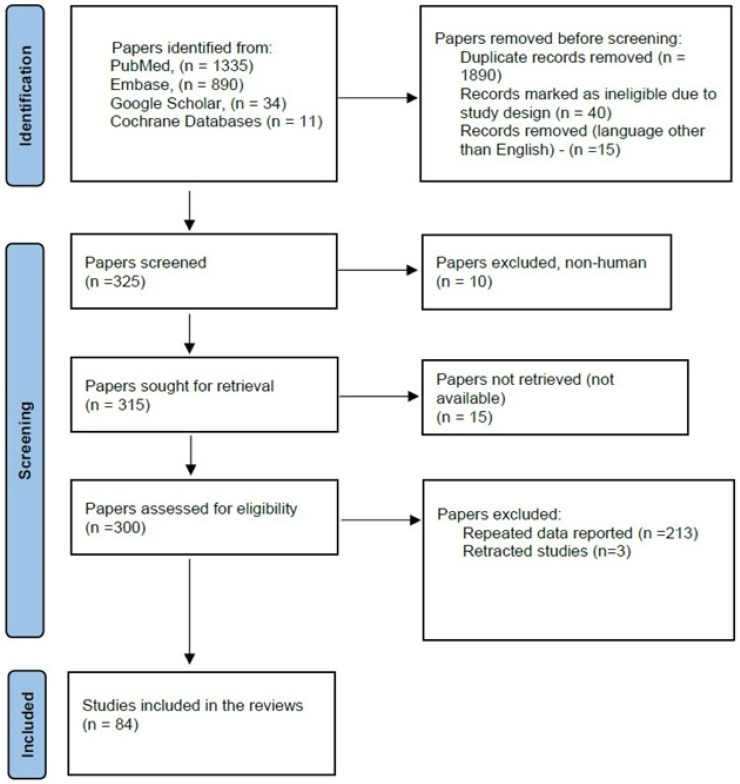
Data search strategy.

A literature search was conducted in the PubMed, Embase, Google Scholar, and Cochrane databases from January 2020 to December 2024. The search was performed using the following keywords: “cervical cancer”, “human papillomavirus”, “HPV”, “premalignant cervical lesions”, “high-risk HPV”. Medical subject heading (MeSH) terms were used whenever available: “human papillomavirus” (MeSH Unique ID: D000094302) as a major topic, “uterine Cervical Neoplasms” (MeSH Unique ID D002583). The search was specified and targeted by using “cervical cancer” OR “uterine Cervical Neoplasms”, AND “premalignant cervical lesions” AND “human papillomavirus”, OR “HPV”, AND/OR “high-risk HPV”. Studies reporting on the global prevalence of HPV, and the persistence and progression of HPV infection, including systematic reviews and meta-analyses published in English, were included. In total, over 2270 articles were reviewed, of which 2186 studies were excluded, leaving 84 articles for further analysis (Figure 1).

Exclusion criteria: articles published in languages other than English prior to January 2020 and not meeting the keywords in the search strategy. Titles and abstracts of articles were retrieved by applying the search strategy and investigated by the authors to categorize samples that could potentially meet the aims of this review. Duplicated studies and irrelevant articles that did not fulfill the listed search criteria were excluded. Full texts of these hypothetically eligible studies were retrieved and evaluated for suitability. Peer-reviewed articles published in English and discussing HPV infection, HPV type, cervical cancer, and precancerous cervical lesions were included in this review.

### 2.2. Review Approach and Justification

A narrative review approach was selected for this manuscript to provide a comprehensive synthesis of the existing literature on the impact of HPV on cervical lesion development and persistence. This review aims to integrate findings from diverse sources, covering multiple dimensions of HPV research, including epidemiology, molecular mechanisms, interactions with the cervicovaginal microbiota, advancements in diagnostic biomarkers, screening strategies, and psychological aspects associated with HPV infection. Given the breadth and complexity of these interconnected topics, a narrative review allows for a more flexible and in-depth discussion that contextualizes findings within the broader scientific landscape.

Unlike systematic or scoping reviews, which adhere to predefined protocols for study selection, data extraction, and analysis, a narrative review is particularly well-suited for exploring multifaceted subjects where the integration of knowledge across disciplines is essential. The decision to use this approach was based on the need to synthesize heterogeneous data from various study designs, including epidemiological surveys, molecular research, systematic reviews, meta-analyses, and clinical reports. A systematic or scoping review methodology would require strict inclusion criteria and a narrower research focus, potentially limiting the scope of discussion and excluding relevant insights from emerging areas of research.

By adopting a narrative approach, this review highlights key trends, knowledge gaps, and evolving perspectives on HPV-related disease progression and prevention strategies. The inclusion of diverse sources enables a holistic understanding of the topic, offering valuable insights for clinicians, researchers, and public health professionals. Furthermore, this format allows for a critical examination of findings, identifying inconsistencies across studies and proposing directions for future research. Given the dynamic nature of HPV research, where new diagnostic and preventive measures are continuously being developed, a narrative synthesis provides the necessary flexibility to incorporate and analyze recent advancements.

## 3. Results and Discussion

### 3.1. Molecular Mechanisms of HPV-Driven Cancerogenesis

HPV is the primary etiological factor in the development of cervical cancer [10,11,12,13,14,15,16,17,18,19]. The genotypic distribution of HPV varies by geographic region, which is critical for developing effective prevention and treatment strategies and combating HPV-associated diseases [12,13,14].

A systematic review and meta-analysis conducted in 2020 examined the impact of HPV-16 and HPV-18 on survival rates of cervical cancer patients [12]. It was found that HPV-18 infection is associated with poorer overall, recurrence-free, and progression-free survival compared to HPV-16, which does not significantly influence survival outcomes. Furthermore, the survival rates of patients with HPV-18 were worse than those with HPV-16 [12,13,22,23,24,25,26,27,28,29,30]. This meta-analysis confirmed that only HPV-18 is linked to poorer survival outcomes [12].

HPV-6 and HPV-11 are traditionally considered low-risk types primarily associated with benign lesions, such as genital warts. However, a systematic review identified cases where these HPV types were linked to the development of malignant tumors [31]. The prevalence of HPV-6 and HPV-11 mono-infections in cervical cancer is minimal (~1–4%), consistent with their low oncogenic potential. However, the similarity of E7 oncogenes may explain their association with carcinomas outside the cervix. The authors emphasize the need to revise current perceptions of HPV-6 and HPV-11 pathogenicity and recommend incorporating these findings into cancer prevention strategies [31].

The E2 protein of HPV-16 plays a critical role in regulating viral replication and the expression of the oncogenic proteins E6 and E7 (Table 1). Studies have demonstrated that E2 can induce apoptosis in infected cells, suggesting its potential as a therapeutic target in HPV-associated cancer treatment [32]. The authors also note that the E2 protein of high-risk HPV types localizes both in the nucleus and the cytoplasm, activating apoptosis. In contrast, in low-risk types like HPV-6 and HPV-11, E2 remains confined to the nucleus and does not trigger apoptosis [32]. Moreover, experiments revealed that the E2 protein can enhance apoptosis under the influence of steroid hormones and radiation therapy. This systematic review summarizes the mechanisms through which E2 induces apoptosis and highlights the potential of utilizing this protein as a therapeutic agent against cancer cells infected with HPV-16 [32,33,34,35,36,37].

### 3.2. Prevalence of HPV in the World Regions

One of the world regions with the highest prevalence of the infection is Sub-Saharan Africa. A systematic review and meta-analysis conducted in 2024 examined the prevalence of HPV in Ethiopia [15]. The authors reported that among women with precancerous cervical lesions in medical facilities, the prevalence of any HPV genotype was 38.75%. Among women with atypical cells (ASCUS and LSIL) and precancerous lesions, HPV was detected in 13.7–93% of cases. The prevalence of HPV-16 (9.52%) and HPV-18 (8.33%) was lower than that of other types, such as HPV-51 (16%) and HPV-52 (13%). The researchers also compared these findings with data from other countries. In Africa, the prevalence of HPV-16/18 among high-grade lesions is 45.1%, while among atypical cytological results, it reaches 67.7%. In Asia, HPV-16 is the most prevalent type (23.9%), which aligns with data from Ethiopia. Similarly, HPV-16 dominates in North America and Europe, whereas in Ethiopia, HPV-51 and HPV-52 are more frequently observed [15].

In a meta-analysis conducted in the Middle East and North Africa (MENA) region, the overall HPV prevalence in the clinical subgroup of cervical cancer cases was reported at 81% [16]. The highest prevalence was observed in North Africa, while the lowest rates were recorded in the Gulf Cooperation Council (GCC) countries and Yemen. The Levant and GCC regions were the least affected. Middle-income countries demonstrated higher HPV prevalence rates compared to low-income countries [16]. The most common HPV genotypes found in cervical cancer samples were HPV-16 and HPV-18. Morocco reported the highest prevalence of HPV and cervical cancer mortality, followed by Turkey and Algeria. Conversely, Kuwait and Iraq showed the lowest incidence and mortality rates [16]. Approximately 50% of cases with abnormal cervical cytology results were associated with HPV infection, with the highest rates observed in North Africa and the lowest in the Levant and Turkey. In summary, the prevalence of HPV in cervical cancer cases in the MENA region is comparable to that of Africa (86.5%) and Asia (79.3%) but lower than in North America and Australia. Among the general population, HPV prevalence in the MENA region is 16%, which is higher than the global average of 10–11% [16].

A systematic review in the Asia-Pacific region revealed a high incidence and prevalence of HPV-associated diseases in Southeast Asia and the Western Pacific [17]. The incidence of cervical cancer ranged from 15.7 to 252 cases per 100,000 person-years, with the highest rates reported among HPV-positive women in Taiwan. The most prevalent HPV types in the region were HPV-16, -18, -45, and -52 [17].

According to two studies, including a 2020 meta-analysis [18] and a 2024 systematic review [19] conducted in China, the most common HPV genotypes among women with CIN are HPV-16, -52, -58, -33, and -18. In the CIN1 group, HPV-52 and -58 are predominant, while in the CIN2/3 group, HPV-16 dominates, followed by HPV-58 and -52. The authors highlight that the distribution of HPV genotypes in China differs from other regions worldwide, necessitating the adaptation of vaccination and screening strategies [18,19]. In China, HPV-16, -52, and -58 are the most prevalent, whereas other regions, such as Europe, North America, and Africa, report different genotype combinations, such as HPV-33 and -45 [18,19].

A systematic review conducted in Korea identified the predominant HPV types as HPV-16 (19.4%), HPV-58, HPV-53, HPV-52, and HPV-18 [22]. HPV-16 is the most dominant type in severe lesions (HSIL and above), while HPV-18 and HPV-58 are also common in HSIL+ cases. HPV-58 is detected across a wide range of lesions, including normal cytology (10.1%), ASCUS (11.4%), LSIL (11.0%), and HSIL (17.0%) [22]. Persistent infections with HPV-16 and HPV-58 pose the highest risk of progression to HSIL, with hazard ratios of 5.04 and 5.84, respectively. For progression to CIN2+, the most oncogenic types include HPV-16, -33, -31, -45, and -18 [22]. The authors also note that the prevalence of HPV-53 and HPV-58 in Korea is higher than in other Asian countries. In contrast, HPV-16, -58, and -82 are prevalent in the Eastern Mediterranean region, while HPV-45 dominates in sub-Saharan Africa [22].

In Japan, the prevalence of any HPV type among women with normal cytology was 15.6%, while it was 86.0%, 76.9%, and 75.7% in cases of HSIL, CIN3/AIS, and invasive cervical cancer, respectively [12]. HPV-16 and HPV-52 are predominant in squamous cell carcinoma, whereas HPV-16 and HPV-18 are more common in adenocarcinoma. HPV-52 and HPV-58 are detected more frequently in Japan compared to other regions of the world [23]. The peak HPV prevalence among women with normal cytology occurs at ages 20–29, followed by a decline [23].

A 2024 systematic review revealed a high overall prevalence of HPV (85%) among patients with cervical cancer in India. The most common genotypes were HPV-16 (58%) and HPV-18 (16.5%) [24].

In Saudi Arabia, a systematic review conducted in 2024 identified a significant correlation between HPV infection and the development of cervical cancer [28]. Additionally, a link was found between HPV integration and ovarian cancer, providing insights into the disease’s etiology. The authors highlight that molecular studies could refine the understanding of HPV-related carcinogenesis mechanisms and support the development of personalized treatment methods. They recommend further research into HPV’s role in the development of various cancer types, which could open new opportunities for targeted therapies and a personalized approach to treatment [25].

The prevalence of HPV in Brazil is high and varies across regions and anatomical sites, with the highest frequencies observed in the penis, anal region, and cervix [26]. HPV prevalence in Brazil is higher than in other regions of the world, such as Central America (13%), North Africa (9.2%), Western Europe (9%), and South Asia (7.1%). The highest prevalence of HPV is found in penile cancer (36%), followed by cervical and anal cancers (25% each), and the oral cavity (12%). HPV prevalence in the cervix is 24.11% among low-risk women and 38.01% among high-risk women. In northern Brazil, which has the highest incidence of cervical cancer, HPV prevalence was not higher, potentially due to limited access to screening programs. HPV-16 and HPV-18 are the most common genotypes; however, Brazil has a relatively high prevalence of HPV-18 compared to other countries [26].

The significant impact of HPV on the risk of cervical cancer among women in Nigeria has been confirmed by a systematic review and meta-analysis, emphasizing the need for targeted preventive measures, including vaccination and screening [27]. These findings can serve as a foundation for developing effective national programs to combat HPV and cervical cancer. The overall prevalence of HPV among women in Nigeria was 25%, varying by region: 45% in the southwest, 70% in the northeast, 76% in the northwest, and 16% in the central region. Among women living with HIV, HPV prevalence reached 71%, which is higher than in other low- and middle-income countries (51%). The most common HPV genotypes were HPV-18 (10%), HPV-16 (7%), HPV-35 (7%), and HPV-58 (7%). Notably, HPV-18 was more prevalent in the study population, differing from trends observed in other countries [27].

A systematic review and meta-analysis were conducted to determine the prevalence of high-risk HPV (HR-HPV) genotypes in Sub-Saharan African countries, examine their association with HIV, and assess the effectiveness of current vaccination programs [28]. It is widely recognized that women living with HIV have a higher prevalence of HPV and an increased risk of cervical cancer compared to HIV-negative women, even with the use of antiretroviral therapy. For example, a study from South Africa found notable differences in HPV prevalence between HIV-negative and HIV-positive women, with rates of 19.1% (71/373) and 45.4% (153/337), respectively, for HPV types 16, 18, 45, 31, 33, 52, 58, and 35 [28,29]. The most common HR-HPV genotypes in the region include HPV-16, -18, -31, -33, -35, -45, -52, -56, -58, and -59, which are the primary oncogenic types. HPV-16 remains the dominant genotype (18%), followed by HPV-35 (10.12%) and HPV-52 (9.98%). Genotype distribution varies within the region and across countries; for instance, HPV-35 and HPV-52 are more prevalent in Chad and Mozambique [28,30].

Another study conducted in 2021 examining racial disparities in HPV prevalence between Asia and Africa highlighted that cervical cancer incidence is influenced by HPV prevalence, public awareness, sexual behavior, vaccination, and screening (Table 2) [31]. In Africa, HPV vaccination rates are low (1.2–4.1%), and knowledge of risk factors and willingness to vaccinate are significantly lower compared to Asia. The most prevalent genotypes in Asia are HPV-16, HPV-52, and HPV-58, while in Africa, HPV-16, HPV-18, and HPV-52 dominate. HPV-35 and multiple infections are more common in Africa, contributing to a higher cervical cancer burden. South Africa has the highest prevalence of both single and multiple infections, whereas Central Africa has the lowest. The authors also suggest that Northern and Eastern Africa have better prospects for eradicating cervical cancer due to greater access to vaccination. In contrast, Central, Western, and Southern Africa face challenges due to the high prevalence of non-vaccine genotypes, complicating efforts to combat the disease [31].

Globally, HPV types 16 and 18 are the most prevalent in both LSIL and HSIL cases. A study from Southern Mexico reported that HPV 16 was the most commonly identified genotype in both cervical cancer and HSIL cases, while HPV 33 was the most frequent in LSIL and non-intraepithelial lesions. In China, a study conducted in Taizhou City found that among women with LSIL, the most common HPV genotypes were HPV 16, 58, 53, and 56. In HSIL cases, HPV 16, 52, 58, 33, and 31 were most prevalent. The study highlighted regional variations, noting that HPV 16 was the most common genotype in almost all age groups for different cervical lesions, except in the 50–59 years age group for LSIL, where it ranked second [32]. In Europe, HPV 16 and 18 are also the most common types found in both LSIL and HSIL cases. A study from the Netherlands reported that among women with HSIL, 98.3% were infected with high-risk HPV, with HR-HPV16 being the most common type, followed by HR-HPV33 [33]. In Africa, the prevalence of HPV types in LSIL and HSIL cases varies. A study from South Africa found that HPV 33 was the most frequent in LSIL cases, while HPV 16 and 18 were more common in HSIL cases [34]. These regional variations underscore the importance of understanding local HPV type distributions to inform effective screening and vaccination strategies.

### 3.3. HPV and Cervico-Vaginal Microbiota

**Figure 2:**
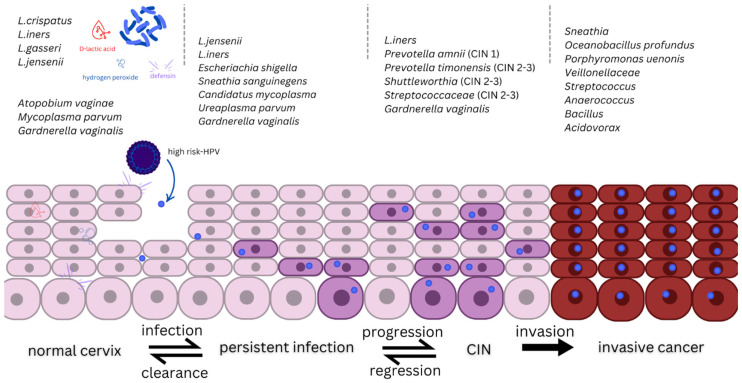
Cervico-vaginal microbiota and HPV infection persistence.

The microbiota of the female reproductive tract plays a crucial role in maintaining health and protecting against infections, including HPV. Disruption of the microbiota balance, known as dysbiosis, can increase susceptibility to infections and lead to adverse reproductive outcomes. Studies suggest that the microbiota contributes to HPV protection and reduces the risk of its persistence (Figure 2) [35,36,37,38,39,40,41,42,43,44,45]. Restoring microbiota balance through probiotics and other therapeutic approaches offers promising prospects for preventing and treating infections [35,36,37,38,39,40,41,42,43,44,45].

A systematic review identified key bacteria associated with HPV and cervical cancer among Latina women [45]. Latina women with dysplasia and cervical cancer exhibit increased alpha and beta diversity in their microbiome. The levels of *Lactobacillus* spp. are lower among Latina women compared to other ethnic groups. *Lactobacillus crispatus* is associated with a healthy microbiome and resistance to HPV, whereas *Lactobacillus iners* is more frequently detected in cases of HPV infection, dysplasia, and bacterial vaginosis (Table 3). Other bacterial species such as *Chlamydia trachomatis*, *Prevotella* spp., *Fusobacterium* spp., and *Sneathia* spp. are linked to HPV infection, dysplasia, and cervical cancer [45].

A study published in 2023 demonstrated that women with bacterial vaginosis have a significantly higher risk of persistent infection with oncogenic HPV strains [46]. An increase in anaerobic bacteria, such as *Gardnerella*, *Prevotella*, *Atopobium*, and *Sneathia*, is associated with HPV infection and its progression to cervical cancer.

The role of *Lactobacillus iners* remains controversial, as it may either protect against or contribute to the progression of HPV infection. Anaerobes promote oxidative stress, reduce levels of protective *Lactobacillus*, and facilitate the progression of preneoplastic changes. *Sneathia* and *Atopobium* can damage epithelial tissues, enhancing susceptibility to HPV infection and cancer development [46]. These findings underscore the importance of maintaining a healthy microbiota balance to prevent chronic HPV infection.

The microbiota also plays a crucial role in modulating the local immune response. A study published in Frontiers in Oncology highlighted that disruptions in microbiota composition can impair the function of antigen-presenting cells, weakening the immune response to HPV [47].

HPV evades the host immune response by disrupting both innate and adaptive immunity, contributing to its persistence and the development of cervical cancer. Women infected with high-risk HPV types show increased proportions of *Bacteroidetes* and *Fusobacteria*, as well as a strong correlation with bacterial vaginosis, *Ureaplasma urealyticum*, and *Chlamydia trachomatis* infections [47].

The presence of dysbiosis can promote chronic inflammation, creating favorable conditions for infection progression by inducing inflammation, increasing cytokine levels, disrupting cervical mucus, and damaging the epithelium [48].

Bacterial enzymes, such as sialidase, degrade the protective epithelial layer, facilitating HPV penetration and integration into the cells of the cervical transformation zone. Anaerobic metabolism in bacterial vaginosis produces ammonia and ammonium nitrite, which have carcinogenic potential and may contribute to cellular changes. Maintaining a healthy microbiota composition is considered a promising approach for preventing and treating HPV infections. The use of probiotics containing lactobacilli has shown positive results in restoring the microbiota and reducing the risk of HPV persistence [48].

Despite significant progress, research on the interaction between the microbiota and HPV remains limited. A 2023 review highlights the need to standardize methods for microbiota analysis and investigate the effects of various bacterial species on the immune response [49]. Additionally, the influence of ethnicity on microbiota and HPV infection is not fully understood. Women of Afro-Caribbean origin exhibit microbiota alterations, although their HPV prevalence was not significantly higher, whereas studies in African populations report higher HPV prevalence [49]. The review also emphasizes the importance of studying the long-term effects of probiotics and other microbiota-modifying interventions for preventing and treating HPV infections.

A 2022 meta-analysis did not find a significant association between *C. trachomatis* infection and the development of cervical cancer [50]. While some studies suggest a potential link, the results remain inconclusive, likely due to factors such as co-infection with HPV, methodological differences, and population variations.

However, co-infection with *C. trachomatis* and HPV may increase the risk of cervical cancer, particularly in cases of persistent HPV infection. *C. trachomatis* promotes chronic inflammation, DNA damage, and immune suppression, facilitating HPV entry and persistence in cervical epithelial cells [50].

Trichomonas vaginalis also plays a significant role in modulating the impact of HPV. A meta-analysis involving data from 150,605 women found that the presence of *T. vaginalis* increases the risk of HPV persistence and infection progression by disrupting the epithelial barrier and amplifying local inflammation [51].

Additionally, *T. vaginalis* is associated with higher HPV viral loads, which may elevate the risk of infection transmission. A healthy microbiota dominated by lactobacilli helps maintain an acidic environment that inhibits the growth of pathogenic microorganisms [50]. Data on HPV prevalence among women of reproductive age and its impact on pregnancy outcomes highlight the need for detailed investigation. HPV infection is associated with an increased risk of adverse pregnancy outcomes, underscoring the importance of this issue [52,53,54,55].

Systematic reviews and meta-analyses investigating the impact of HPV-16/18 on premature rupture of membranes (PROM) and preterm birth have established that women at high risk of HPV infection are more likely to experience PROM and preterm delivery [52,54,55]. The infection contributes to matrix degradation of the fetal membranes and prostaglandin activation, which stimulates uterine contractions. Additionally, HPV weakens the cervical immune defense, increasing the risk of inflammation and preterm birth. The authors conclude that HPV screening before pregnancy should be considered and incorporated into standard medical examinations [52,54,55]. However, some studies report no significant association between HPV infection and spontaneous miscarriage or gestational hypertension [53,55].

### 3.4. HPV and Premalignant Cervical Lesions: Modern Diagnostic Biomarkers

A systematic review conducted in 2020 evaluated the effectiveness of mRNA E6/E7 tests in detecting CIN2+ lesions. The results demonstrated that these tests have high specificity compared to HPV DNA tests, which can reduce false-positive results and unnecessary interventions [38]. Similarly, a 2024 meta-analysis confirmed the high specificity of E6/E7 oncoprotein tests (over 82%) across all groups, regardless of HPV or HIV status, underscoring their potential as a diagnostic tool [39].

A 2024 meta-analysis in China compared the diagnostic value of mRNA E6/E7 tests with traditional methods, such as cytology and HPV DNA testing. The findings revealed that mRNA E6/E7 tests have higher specificity with comparable sensitivity, making them preferable for screening programs to minimize false-positive results [40]. Another study assessed the use of serological markers for E6 and E7 oncoproteins of high-risk HPV types in diagnosing cervical cancer. The results indicated that antibodies to HPV16/18 E6 and E7 oncoproteins have high specificity but low sensitivity for diagnosing cervical cancer and precancerous lesions (CIN2/3) [41].

Despite advancements in diagnostics, the implementation of screening methods is hindered by financial and infrastructural limitations. The primary screening methods for cervical cancer and precancerous lesions remain the Pap smear and high-risk HPV testing. However, their limited accuracy in predicting the progression of lesions such as CIN2/3 to cancer highlights the need for additional biomarkers [43].

Studies of new markers, such as p16 and hTERC, in combination with traditional methods, have the potential to improve the diagnosis and prognosis of cervical cancer, enabling personalized treatment and reducing unnecessary medical interventions. Dual staining with p16/Ki-67 (DS) has emerged as a promising tool, providing high sensitivity (up to 93%) and specificity (up to 60%) for detecting CIN2+ in women with ASC-US and LSIL, thereby reducing the number of unnecessary colposcopies. The combination of p16 with HPV testing shows a sensitivity of 89.58% and specificity of 72.73%, making this approach particularly valuable in resource-limited settings [43].

hTERC gene amplification has demonstrated high efficacy as a predictor of precancerous lesion progression. When combined with cytology and HPV testing, this marker achieves a sensitivity of up to 100% and specificity of up to 98.11% [34]. Additionally, fibronectin, which plays a role in cell migration and oncogenesis, is being considered as a potential biomarker for cervical cancer. However, further research is needed to validate its clinical applicability [43].

DNA methylation and microRNA expression levels play a crucial role in regulating oncogenes and tumor suppressor genes in HPV-related oncogenesis, contributing to genetic instability and disease progression [42]. The FAM19A4/miR124-2 test demonstrates high accuracy (up to 98%) in diagnosing cervical cancer (CC) and CIN2/3 lesions. A negative test result effectively excludes progressive lesions, minimizing the risk of unnecessary treatment. MicroRNAs such as miR-124-2 and miR-375 are significant prognostic indicators for CIN and CC, aiding in assessing disease progression and treatment efficacy. Combining DNA methylation analysis, microRNA profiling, and HPV genotyping significantly enhances diagnostic precision and supports the implementation of personalized approaches in patient management and treatment [42].

Modern biomarkers significantly enhance the diagnosis and prognosis of cervical cancer (CC) and precancerous conditions [44]. Among the most studied biomarkers are HPV E6/E7 mRNA, miR-9, p16INK4a/Ki-67, DNA methylation, SCC-Ag, M-CSF, and VEGF. HPV E6/E7 mRNA is associated with CIN2+ progression, showing sensitivity ranging from 65–100% and specificity from 42.7–90.2%, although further validation is required. MicroRNA miR-9, involved in transcriptional regulation, demonstrates a sensitivity of 52.9–67.3% and a specificity of 76.4–94.4%, making it valuable for early diagnosis, prognosis, and treatment monitoring. Its utility is especially pronounced when combined with other microRNAs, such as miR-21 and miR-155 [44].

The combination of p16INK4a/Ki-67 provides high sensitivity (up to 100%) and specificity (up to 90.4%) for detecting CIN2+, enabling a reduction in unnecessary colposcopies and expediting diagnosis. This test can serve as an alternative to HPV DNA testing in resource-limited settings. DNA methylation of key genes, such as JAM3 and SOX1, is associated with CIN2+/CIN3 and demonstrates sensitivity ranging from 59.7–92.9% and specificity from 67–98%, further enhancing diagnostic accuracy for cervical lesions [44].

The SCC-Ag marker and macrophage colony-stimulating factor (M-CSF) are associated with the progression of CIN2+/CIN3 and tumor aggressiveness, while VEGF, which plays a role in angiogenesis, achieves a sensitivity of 83.5% and specificity of 96%. These markers effectively complement traditional diagnostic methods, enhancing accuracy and prognostic capabilities [44].

A 2020 systematic review confirmed that p16INK4a/Ki-67 and DNA methylation are among the most promising markers for triaging women with positive HPV test results (Table 4) [44]. The addition of miR-9 and VEGF enhances diagnostic and prognostic accuracy while reducing the risk of unnecessary medical interventions [44]. Another study from China demonstrated that ZNF582 methylation exhibits high diagnostic accuracy for detecting CIN3+. Combining HPV DNA testing with ZNF582 methylation tests significantly improves diagnostic efficiency compared to standalone HPV testing. The authors suggest considering ZNF582 methylation as an additional biomarker for cervical cancer screening [56,57,58,59,60].

HPV DNA testing has become the “gold standard” for primary screening in several European countries at both regional and national levels. However, this approach increases the burden on screening programs, as the number of referrals for colposcopy rises significantly due to the limited specificity of HPV tests, which cannot distinguish transient infections from progressive lesions [58]. In recent years, DNA methylation biomarkers have been investigated for triaging hrHPV-positive cases to reduce the number of colposcopy referrals and lower the rates of overdiagnosis and overtreatment [58]. Despite this, DNA methylation has shown comparable specificity to cytology (using ASC-US as the threshold) for detecting CIN2+, though its sensitivity was slightly lower. For CIN3+, methylation demonstrated higher specificity with similar sensitivity compared to cytology [58]. Studies have shown that a negative DNA methylation test was associated with a higher likelihood of regression of high-grade lesions (CIN2/CIN3). In some cases, combining a negative methylation test with negative HPV16/18 genotyping increased the probability of regression to 85% [58].

A 2022 systematic review and meta-analysis reported that diagnostic inaccuracies of colposcopy-directed cervical biopsy were more pronounced in women aged 50 and older. Postmenopausal status and type 3 transformation zone were also associated with a higher likelihood of diagnostic errors. Diagnostic performance was higher for high-grade lesions (HSIL) compared to low-grade lesions (LSIL) [59]. This meta-analysis demonstrated that the correlation between histological biopsy results and findings after surgical treatment depends on the woman’s age, menopausal status, and the type of transformation zone [59].

A systematic review and meta-analysis involving 2817 patients demonstrated that the folate receptor-mediated diagnostic test (FRD) has good overall diagnostic accuracy for detecting CIN2+ lesions [61]. Based on this study, FRD appears to be a promising tool for cervical cancer screening.

### 3.5. HPV and Cervical Cancer Screening: Updated Approach

Although cervical cancer is a preventable disease, it remains a significant public health challenge worldwide. The elimination of cervical cancer largely depends on screening programs and vaccination efforts. The World Health Organization (WHO) recommends HPV testing as one of the most effective screening methods for cervical cancer. HPV genotyping for primary cervical cancer screening offers significant advantages for risk stratification, enhancing the effectiveness of screening programs. Here’s an overview of how extended genotyping can improve outcomes:

Extended genotyping enables the identification of specific HPV genotypes with varying oncogenic potential. While HPV 16 and 18 pose the highest risk for cervical cancer, other types such as 31, 33, 45, 52, and 58 also contribute significantly. Differentiating these high-risk strains from lower-risk ones allows clinicians to more accurately assess a patient’s risk and customize follow-up and treatment strategies.

Identifying the specific HPV type can influence clinical management, determining the urgency and nature of follow-up care. Women with high-risk HPV infections may require immediate and intensive monitoring, including colposcopy and biopsy, while those with lower-risk types can be managed with extended-interval repeat testing.

By stratifying patients based on their likelihood of progression to cervical cancer, extended genotyping helps allocate healthcare resources more efficiently. This targeted approach reduces unnecessary interventions for low-risk individuals, minimizing costs and the risks associated with overtreatment [32,33,34,35,36,37,38,39,40,41,42,43,44,45,46,47,48,49,50,51,52,53,54,55,56,57,58,59,60,61,62,63,64].

Integrating extended genotyping into national and regional cervical cancer screening protocols enhances their effectiveness. It enables personalized screening intervals and ensures that high-risk individuals receive timely intervention, thereby improving overall prevention strategies.

Data derived from extended genotyping provide valuable insights into the distribution and prevalence of different HPV types across populations. This information supports public health initiatives, informs policy decisions, and aids in evaluating and refining HPV vaccination programs for maximum impact [62,63,64].

An increasing body of evidence supports the use of self-sampling as an alternative to clinician-collected samples for screening. Self-sampling can improve coverage among women who have not participated in screening for extended periods, making it a promising strategy for low- and middle-income countries. Easy-to-use self-testing devices have the potential to reduce cervical cancer mortality and morbidity [65].

Self-sampling methods for HPV DNA testing, as well as visual inspection with acetic acid or Lugol’s iodine, have been proposed as alternative screening approaches for women in resource-limited settings. Self-sampling for HPV DNA testing showed high acceptability among patients; however, differences in accuracy between self-sampling and clinician-collected samples remain unclear. A systematic review conducted in Kazakhstan in 2024 compared various types of self-sampling devices to identify the most accurate and acceptable options. According to the findings, devices such as the Evalyn Brush (Rovers Medical Devices, Amsterdam, The Netherlands), Cervex-Brush (Rovers Medical Devices, Amsterdam, The Netherlands), FLOQ Swab (Copan Diagnotics, Italy), and Delphi Screener (Rovers Medical Devices, Amsterdam, The Netherlands) proved to be the most reliable and acceptable for women. In terms of diagnostic accuracy, self-sampling demonstrated results comparable to the traditional clinician-collected method [65].

Another study conducted by researchers from the University of Catanzaro “Magna Græcia” in Italy during the COVID-19 pandemic highlighted the significant impact of the pandemic on cervical cancer screening attendance. Self-sampling presents a unique opportunity to address gaps in screening programs and increase cervical cancer screening coverage. The WHO strongly recommends self-sampling for HPV testing to achieve 70% screening coverage by 2030 and to eliminate HPV-associated diseases in the coming decades [66]. The meta-analysis revealed that women were nearly twice as likely to participate in screening when self-sampling was offered compared to clinician-collected samples. Furthermore, the accuracy of HPV testing using self-sampling was comparable to that of clinician-collected methods [66]. The high acceptability of self-sampling, particularly among women who had not previously participated in screening, underscores its potential to expand cervical cancer screening coverage and reduce cervical cancer incidence and mortality. However, cultural, religious, and socio-economic factors must be considered when designing self-sampling campaigns. For instance, studies indicate that religious beliefs may reduce women’s participation in self-sampling, particularly in Islamic communities where cervical cancer prevention remains a sensitive topic [66].

The results of another meta-analysis also confirm that self-sampling for HPV is more effective in engaging women than traditional invitation methods, regardless of the invitation strategy used [67]. In the Netherlands, the proportion of women using self-sampling kits increased from 7% in 2017 to 16% in 2020 due to the simplification of the request process [67]. The implementation of HPV self-sampling in Sweden has significantly boosted cervical cancer screening coverage. A study found that after self-sampling kits were distributed to all eligible women in the Stockholm region, the screening rate increased from 75% to 85% within a year. This rise was especially notable during times of disruption to conventional healthcare services, such as the COVID-19 pandemic. The need for alternative screening methods during such periods highlighted the adaptability and effectiveness of self-sampling as a resilient public health strategy in times of crisis [68].

Self-sampling for HPV can significantly increase screening coverage and engagement among underrepresented groups, such as Latina women, as reported in a systematic review conducted in the United States [69]. This approach is particularly valuable for overcoming access barriers related to language, racial discrimination, and difficulties accessing healthcare services. Women reported that self-sampling is convenient, easy to perform, ensures greater privacy, and causes less embarrassment compared to clinical examinations. However, the main drawback was anxiety about performing the procedure incorrectly, leading to a preference for clinician-collected samples. While cervicovaginal self-sampling was generally acceptable, women expressed a preference for alternative sample types, such as saliva, urine, or menstrual blood [69].

A systematic review conducted among Muslim women identified several positive factors contributing to the acceptance of HPV self-sampling. These factors included convenience, cultural appropriateness, and accessibility, which led to higher test acceptability among this population [70]. Self-sampling allowed women to perform the procedure independently without involving medical staff, thereby reducing feelings of discomfort. Participants noted that the test was painless since they had full control over the process. Additionally, the procedure was quick and could be completed by following simple instructions. These characteristics make HPV self-sampling particularly appealing, especially for hard-to-reach populations, offering a culturally sensitive and accessible approach to increasing screening coverage [70].

Another positive factor was the cultural appropriateness of the test. Muslim women viewed self-sampling as a suitable method that ensured complete privacy and modesty, aligning with their religious and cultural values. The test eliminated the need for medical staff involvement, which was particularly important for married women who often needed to consider their spouses’ opinions in such matters. Religious beliefs played a key role in the choice of medical procedures, with an emphasis on maintaining modesty during medical interactions. These aspects made self-sampling an appealing option for cervical cancer screening among Muslim women, addressing their specific cultural and religious concerns [70].

A systematic review and meta-analysis conducted among African women found that HPV self-sampling and clinician-collected samples showed moderate to substantial agreement in results, supporting the feasibility of self-sampling. Among vulnerable populations, the agreement was lower but still significant, indicating its potential applicability [71]. While self-sampling is actively studied and implemented in high-income countries, African countries are still in the early stages of adoption. This highlights the need for further efforts to integrate self-sampling into cervical cancer screening programs in these regions to increase accessibility and participation, particularly among underserved populations [71].

Researchers outlined the main challenges in implementing HPV self-sampling programs in India [72]. Despite the high acceptability of self-sampling in other countries, Indian women are more likely to decline the tests, posing a significant barrier to achieving the 70% screening coverage recommended by the WHO. Women often express concerns about performing the test correctly and providing a quality sample. Additionally, a major socio-economic barrier is the lack of private space for conducting the test, particularly in one-room homes. Studies from Mumbai suggest establishing designated private spaces on campuses where women can collect samples in a confidential setting. Another challenge involves migrants and slum residents who are not registered in official population records, making it difficult to include them in screening programs. In India, sample collection by mobile healthcare workers has been shown to increase screening coverage fourfold, demonstrating a viable approach to overcoming these barriers [72].

A systematic review conducted in Korea demonstrated that HPV testing in urine can detect ≥79% of CIN2 and more severe lesions. PCR-based tests, such as GP5+/6+, SPF10, and others, showed sensitivity comparable to clinician-collected samples (Table 5). However, the sensitivity of urine-based HPV tests was 21% lower in primary screening and 14% lower in follow-up settings compared to tests using clinical samples. The specificity of urine-based tests for excluding CIN2 or more severe lesions was 87% for primary screening and 48% for follow-up, which was 2% and 6% higher, respectively, than clinician-based tests [73]. Sensitivity was slightly higher in low- and middle-income countries, likely due to the higher prevalence of HPV in these regions [73]. Urine-based HPV tests can be particularly useful for specific populations where cultural preferences or barriers reduce participation in clinician-led screening. For instance, in Thailand, women often decline screening due to a lack of symptoms, fear of pain, or embarrassment. Similarly, studies in Korea showed that satisfaction with urine-based HPV tests was higher compared to clinician-based methods, indicating their potential to increase screening participation [73].

Various sample types are used for HPV self-sampling, including cervicovaginal secretions, urine, and methods such as tampons, cotton swabs, and cytobrushes. However, many women feel discomfort with inserting devices into their genital area or worry about using them correctly. To overcome these barriers, menstrual blood collected on pads or cloths has been proposed as a simple, convenient, and comfortable screening tool.

A review of five studies from Asian countries found that the sensitivity of HPV tests using menstrual blood ranged from 82.8% to 97.7%, while specificity ranged from 50% to 98% [74]. Menstrual blood as a sample can increase women’s participation in screening since the procedure does not interfere with daily activities and is suitable for women with hourly jobs [74]. The authors also noted that the day of menstrual blood collection might affect test results. In three out of five studies, it was observed that HR-HPV levels could decrease over the course of menstruation. The highest sensitivity and consistency of test results were reported when testing was performed on the first day of menstruation [74]. Using menstrual blood for screening reduces the costs associated with setting up screening clinics, which can account for 20–40% of total expenses [74].

### 3.6. Psychological Factors in HPV Infection

HPV diagnosis and subsequent treatments, such as loop electrosurgical excision procedures, can significantly affect women’s mental health and sexual function, highlighting the importance of providing comprehensive psychological and emotional support alongside medical care [56,57].

An international study that included data from various countries revealed that women who received a positive HPV test result experienced increased levels of anxiety and psychological distress in the short term. However, in the long term, the differences in anxiety levels between HPV-positive and HPV-negative women diminished (Table 6) [56]. Additionally, women with positive HPV test results but normal cytology also demonstrated heightened anxiety, highlighting the need for psychological support for this patient group [56].

A systematic review conducted by researchers from Poland in 2023 further confirmed that HPV diagnosis and the performance of loop electrosurgical excision procedures (LEEP) could negatively affect women’s mental health and sexual function [57]. The findings underscore the importance of developing support strategies to reduce psychological distress and improve the quality of life for women undergoing such diagnostic and therapeutic procedures [57].

## 4. Strengths and Limitations

Strengths. This manuscript has several notable strengths. First, it provides a comprehensive synthesis of current literature on HPV, covering a broad spectrum of topics, including epidemiology, molecular mechanisms, microbiota interactions, diagnostic advancements, and psychological implications. By integrating findings from diverse sources, including systematic reviews, meta-analyses, clinical studies, and molecular research, it offers a well-rounded perspective on HPV-related cervical lesions and cancer. Second, the review emphasizes regional epidemiological variations in HPV genotype distribution, an essential aspect for tailoring prevention and screening strategies. The discussion of high-risk genotypes beyond HPV-16 and HPV-18, such as HPV-35, HPV-52, and HPV-58, highlights the need for region-specific public health policies and the adaptation of vaccination programs. Third, a major strength of this review is its exploration of emerging diagnostic technologies, including E6/E7 oncoprotein assays, DNA methylation analysis, and microRNA profiling. These biomarkers have the potential to improve screening accuracy by differentiating transient infections from persistent high-risk cases, thereby reducing unnecessary medical interventions. Fourth, the manuscript extends beyond traditional virological perspectives by addressing the role of the cervicovaginal microbiota in HPV persistence and progression. The discussion of microbial dysbiosis, particularly the protective role of Lactobacillus crispatus and the harmful effects of anaerobic bacteria, opens new avenues for potential therapeutic interventions, such as probiotics and microbiota-modulating therapies. Fifth, the review goes beyond biological factors to examine the psychological impact of HPV diagnosis, recognizing the significant distress and stigma associated with a positive HPV test result. Integrating psychosocial support into clinical management is emphasized as an important component of improving patient adherence to screening and treatment. Finally, the discussion of self-sampling methods as a strategy to increase screening accessibility, particularly in resource-limited settings, underscores the potential for reducing disparities in cervical cancer prevention by making screening more widely available.

Limitations. Despite these strengths, the review also has certain limitations. First, as a narrative review, it does not follow the methodological rigor of a systematic or scoping review, meaning that the selection of sources is not based on a predefined systematic protocol. This may introduce selection bias and limit the comprehensiveness of the included literature. Second, while the manuscript discusses findings from various systematic reviews and meta-analyses, it does not perform an independent quantitative synthesis of data. The absence of meta-analytical comparisons means that the review does not provide precise statistical estimations of HPV prevalence, risk factors, or diagnostic accuracy measures. Third, there is potential publication bias, as the review primarily includes peer-reviewed articles published in English, which may exclude relevant research from non-English sources, particularly studies from low- and middle-income countries where HPV burden is high but research dissemination may be limited. Fourth, the inclusion of studies with diverse methodologies, sample sizes, and quality standards introduces heterogeneity in the findings. Differences in study designs, population characteristics, and diagnostic criteria make direct comparisons across studies challenging, potentially limiting the generalizability of conclusions. Fifth, while the review extensively covers HPV prevention, screening, and diagnosis, it offers limited discussion on treatment strategies for HPV-related lesions beyond screening methods. A more detailed examination of therapeutic interventions, including emerging antiviral therapies, immunotherapeutic approaches, and the role of HPV vaccination in preventing post-treatment recurrence, would further strengthen the clinical relevance of the review. Sixth, the review primarily focuses on HPV infection in women and its association with cervical cancer, with minimal discussion on HPV-related diseases in men, such as anogenital and oropharyngeal cancers. Given the rising incidence of non-cervical HPV-associated malignancies, a broader discussion on HPV’s impact beyond the cervix could provide a more comprehensive understanding of the virus’s overall public health implications.

## 5. Conclusions

Human papillomavirus (HPV) remains a major public health challenge due to its well-documented role in the development and persistence of cervical precancerous lesions and cervical cancer. This review underscores the significant regional variations in HPV prevalence and genotype distribution, emphasizing the need for context-specific prevention, screening, and treatment strategies. High-risk HPV types, particularly HPV-16 and HPV-18, continue to dominate the global epidemiological landscape; however, other genotypes, such as HPV-35, HPV-52, and HPV-58, exhibit considerable regional prevalence, which has implications for vaccination and screening programs. The review also highlights the impact of multiple HPV infections, which are associated with increased viral load and persistence, thereby elevating the risk of lesion progression. These findings reinforce the importance of genotype-specific surveillance to inform public health policies and optimize the effectiveness of current HPV vaccines.

This review also highlights the crucial role of advanced diagnostic biomarkers in enhancing HPV detection and cervical cancer screening. While traditional methods like Pap smears and HPV DNA testing are effective, they have limitations in predicting lesion progression and distinguishing transient infections from persistent high-risk cases. Emerging molecular diagnostics—such as E6/E7 oncoprotein assays, p16/Ki-67 dual staining, DNA methylation analysis, and microRNA profiling—offer greater sensitivity and specificity in identifying high-risk lesions. These biomarkers present a promising opportunity to refine screening strategies, minimize unnecessary medical interventions, and enable earlier detection of cervical neoplasia. Additionally, self-sampling techniques for HPV detection have shown high accuracy and acceptability, particularly in resource-limited settings, where they can significantly enhance screening accessibility and reduce barriers to cervical cancer prevention.

Beyond the virological and diagnostic aspects, this review highlights the interplay between HPV infection and the cervicovaginal microbiota, demonstrating that microbial dysbiosis may contribute to viral persistence and progression. The predominance of *Lactobacillus crispatus* in a healthy vaginal microbiome appears to confer a protective effect against HPV, whereas an increase in anaerobic bacteria, such as *Gardnerella*, *Prevotella*, and *Sneathia*, is associated with chronic HPV infection and higher oncogenic risk. These findings open new possibilities for microbiota-targeted interventions, such as probiotics and microbiome restoration therapies, as adjuncts in HPV prevention and treatment.

The psychological and emotional burden of HPV diagnosis further underscores the necessity of integrating psychosocial support into clinical management. The stigma associated with HPV, along with the anxiety and distress following a positive test result or the diagnosis of cervical dysplasia, can have a profound impact on women’s mental health and quality of life. Implementing structured counseling and psychological support programs could help mitigate these effects, improve adherence to follow-up care, and reduce the psychological distress associated with HPV-related conditions.

Directions for Further Research. While significant advancements have been made in HPV research, several areas warrant further investigation. First, ongoing studies are needed to evaluate the long-term effectiveness of next-generation HPV vaccines, particularly in addressing regional genotype variations and improving cross-protection against emerging high-risk types. Second, further research into molecular biomarkers, including the integration of multi-omics approaches such as genomics, proteomics, and metabolomics, could enhance the precision of HPV-related cervical cancer screening and risk stratification. Third, the role of the cervicovaginal microbiota in modulating HPV infection requires deeper exploration, particularly in the context of developing microbiota-based interventions. Additionally, the feasibility of large-scale implementation of self-sampling strategies, including urine- and menstrual blood-based HPV tests, should be further assessed to optimize accessibility and participation rates. Finally, interdisciplinary studies examining the intersection of HPV infection, mental health, and healthcare accessibility could contribute to more holistic prevention and treatment approaches, ultimately improving patient outcomes and reducing the global burden of HPV-associated diseases.

## Figures and Tables

**Table 1:** Molecular mechanisms of HPV-driven cancerogenesis: specific pathways and cellular targets.

Molecular Mechanism	HPV Type	Key Findings	References
E6 oncoprotein and p53 degradation	HPV-16, HPV-18, HPV-31, HPV-33, HPV-45	E6 binds to E6-associated protein (E6-AP), leading to ubiquitin-mediated degradation of p53. This prevents apoptosis, disrupts DNA damage repair, and promotes cellular proliferation.	[4,35]
E7 oncoprotein and Rb inactivation	HPV-16, HPV-18, HPV-31, HPV-33, HPV-45, HPV-52	E7 binds and degrades the retinoblastoma (Rb) protein, releasing E2F transcription factors. This leads to uncontrolled cell cycle progression, increased proliferation, and chromosomal instability.	[23,32]
HPV DNA integration into host genome	HPV-16, HPV-18, HPV-35, HPV-52	Viral integration into fragile sites of the host genome disrupts tumor suppressor genes and leads to uncontrolled oncogene expression. HPV integration often occurs near oncogenes such as MYC, leading to increased tumor aggressiveness.	[23,26]
Loss of E2 gene regulation	HPV-16, HPV-18, HPV-52, HPV-58	HPV integration disrupts the viral E2 gene, which normally inhibits E6 and E7 expression. Loss of E2 leads to sustained overexpression of oncogenic proteins, increasing malignant potential.	[22,32]
Epigenetic modifications (DNA methylation and histone changes)	HPV-16, HPV-18, HPV-31, HPV-58	HPV induces hypermethylation of tumor suppressor genes (e.g., p16INK4a, RASSF1, CADM1, DAPK1) and histone modifications, silencing protective pathways and increasing oncogenesis.	[23,37]
MicroRNA (miRNA) dysregulation	HPV-16, HPV-18, HPV-33, HPV-52, HPV-58	HPV alters host miRNA expression: upregulation of oncogenic miRNAs (miR-21, miR-155) enhances proliferation and invasion, while downregulation of tumor-suppressive miRNAs (miR-375, miR-34a) promotes immune evasion and metastasis.	[25,37]
Oxidative stress and inflammation	HPV-16, HPV-18, HPV-45, HPV-58	HPV-induced chronic inflammation (elevated IL-6, TNF-α, and COX-2) leads to oxidative DNA damage, enhancing genomic instability and malignant transformation.	[23,24]
Telomerase activation (hTERT overexpression)	HPV-16, HPV-18, HPV-33	E6 upregulates human telomerase reverse transcriptase (hTERT), enabling indefinite cell replication, bypassing senescence, and promoting tumor progression.	[4,19]
Cell adhesion and EMT (epithelial–mesenchymal transition)	HPV-16, HPV-18, HPV-45, HPV-58	E6/E7 downregulates E-cadherin and upregulates N-cadherin, vimentin, and Snail, promoting invasion, metastasis, and resistance to therapy.	[23,24]

**Table 2:** Global prevalence and genotypic distribution of high-risk HPV.

Region	Most Common HPV Genotypes	HPV Prevalence in Cervical Cancer Cases	Reference
Sub-Saharan Africa	HPV-16, HPV-18, HPV-35, HPV-52	86.5%	[16,28]
Middle East and North Africa (MENA)	HPV-16, HPV-18, HPV-51	81%	[16]
Asia-Pacific	HPV-16, HPV-18, HPV-45, HPV-52, HPV-58	79.3%	[17,18]
China	HPV-16, HPV-52, HPV-58	High CIN1 prevalence	[18,19]
Korea	HPV-16, HPV-58, HPV-52	85% in HSIL+ cases	[20]
India	HPV-16, HPV-18	85%	[24]
Saudi Arabia	HPV-16, HPV-18	Significant correlation with cervical cancer	[25]
Brazil	HPV-16, HPV-18	25% (cervix), 36% (penile cancer)	[26]
Nigeria	HPV-16, HPV-18, HPV-35, HPV-52, HPV-58	25–76% (regional variation)	[27]
Europe and North America	HPV-16, HPV-18, HPV-31, HPV-33	Highest prevalence of squamous cell carcinoma	[23]

**Table 3:** HPV types and cervico-vaginal microbiota.

Factor	HPV Type	Key Findings	References
Protective microbiota	HPV-16, HPV-18, HPV-58	*Lactobacillus crispatus* dominance is associated with a lower risk of HPV infection and persistence, while its depletion increases susceptibility.	[20]
Microbiota disruptions (dysbiosis)	HPV-16, HPV-18, HPV-52	Increased vaginal microbiota diversity (alpha and beta diversity) correlates with HPV persistence and progression to high-grade lesions.	[15,28]
HPV-associated bacteria	HPV-16, HPV-18, HPV-58, HPV-52	*Lactobacillus iners*, *Gardnerella vaginalis*, *Atopobium*, *Prevotella*, *Fusobacterium*, and *Sneathia* are frequently found in HPV-infected women and those with cervical dysplasia.	[16,19]
Impact on immune response	HPV-16, HPV-31, HPV-33, HPV-45	Dysbiosis weakens the host immune response, facilitating HPV persistence and carcinogenesis. Chronic inflammation caused by *Sneathia* and *Fusobacterium* promotes oncogenic progression.	[23,37]
Mechanisms of HPV persistence	HPV-16, HPV-18, HPV-35, HPV-52	Anaerobic bacteria disrupt cervical mucus, produce carcinogenic metabolites (e.g., nitrosamines), and damage epithelial integrity, aiding HPV integration.	[4,26]
Probiotics and microbiota restoration	HPV-16, HPV-18, HPV-58, HPV-52	Probiotic therapies (e.g., *Lactobacillus crispatus* supplementation) may prevent HPV persistence, modulate the immune response, and restore vaginal microbiota homeostasis.	[25,32]
Ethnic and regional variations	HPV-16, HPV-18, HPV-52, HPV-58, HPV-35	Latina and Afro-Caribbean women have distinct microbiome patterns that may influence HPV infection outcomes. Higher prevalence of *L. iners* and anaerobes correlates with persistent HPV in these populations.	[23,24]

**Table 4:** Diagnostic biomarkers for HPV-related cervical lesions.

Biomarker	Diagnostic Role	Sensitivity/Specificity	Reference
HPV DNA testing	Detects presence of high-risk HPV	High sensitivity, lower specificity	[38]
E6/E7 oncoprotein test	Identifies active HPV-driven oncogenesis	Sensitivity: 82%, specificity: high	[39]
p16/Ki-67 dual staining	Detects high-risk lesions (CIN2+)	Sensitivity: 93%, specificity: 60%	[43]
hTERC gene amplification	Predicts lesion progression	Sensitivity: 100%, specificity: 98.11%	[44]
DNA methylation (FAM19A4/miR-124-2 test)	Identifies CIN2+/CC lesions	High specificity (98%)	[43]
MicroRNA panels (miR-9, miR-21, miR-375)	Prognostic indicators for CIN and CC	Sensitivity: 52.9–67.3%, specificity: 76.4–94.4%	[58]

**Table 5:** Updated approach to HPV and cervical cancer screening.

Screening Method	HPV Type	Key Findings	References
HPV DNA testing (primary screening)	HPV-16, HPV-18, HPV-31, HPV-33, HPV-52	The WHO recommends HPV DNA testing as the most effective primary screening tool due to its high sensitivity. It detects high-risk HPV (hrHPV) before cytological abnormalities appear.	[28,38]
E6/E7 oncoprotein testing	HPV-16, HPV-18	E6/E7 mRNA tests have higher specificity than DNA tests, reducing false positives and unnecessary colposcopies. Studies confirm over 82% specificity across all patient groups.	[39,40]
p16/Ki-67 dual staining	HPV-16, HPV-18, HPV-31, HPV-33	Used for triaging HPV-positive women, this test improves specificity in detecting CIN2+ lesions. It is particularly valuable in ASC-US/LSIL cases, reducing unnecessary colposcopies.	[23,43]
hTERC gene amplification	HPV-16, HPV-18, HPV-33	A predictor of precancerous lesion progression. Combining hTERC with cytology and HPV testing improves sensitivity (up to 100%) and specificity (98.11%) for CIN2+.	[42,44]
DNA methylation biomarkers	HPV-16, HPV-18, HPV-52, HPV-58	FAM19A4/miR124-2 and ZNF582 methylation tests show high specificity (~98%) for detecting CIN2+/CIN3 lesions and are effective for triaging HPV-positive women.	[23,42]
Self-sampling for HPV testing	HPV-16, HPV-18, HPV-31, HPV-52	Self-sampling increases screening participation, particularly in low-resource settings. Devices such as Evalyn^®^ Brush, FLOQSwabs^®^, and Delphi Screener demonstrate diagnostic accuracy comparable to clinician-collected samples.	[23,24]
Urine-based HPV testing	HPV-16, HPV-18, HPV-58	PCR-based urine tests (GP5+/6+, SPF10) detect ≥79% of CIN2+ cases. While useful, they have 21% lower sensitivity in primary screening compared to cervical samples.	[15,19]
Menstrual blood-based HPV testing	HPV-16, HPV-18, HPV-33, HPV-52	Testing menstrual blood for HPV provides a cost-effective alternative with sensitivity ranging from 82.8% to 97.7%. Diagnostic accuracy is highest when collected on the first day of menstruation.	[23,26]
AI-assisted cervical cytology	HPV-16, HPV-18, HPV-31, HPV-33	AI-based Pap test analysis improves detection of precancerous lesions, reducing false negatives and human errors. Digital cytology aids in early cancer diagnosis.	[24,42]
Cultural influences on HPV screening uptake	HPV-16, HPV-18, HPV-31, HPV-52	Religious and cultural beliefs influence self-sampling acceptance. In Muslim communities, self-collection is preferred due to privacy concerns, improving screening uptake.	[23,25]
Strategies to increase screening accessibility	HPV-16, HPV-18, HPV-33, HPV-45	Self-sampling and mobile health workers improve coverage in low-income areas. WHO aims for 70% screening coverage by 2030 to eliminate cervical cancer.	[24,42]

**Table 6:** Psychological factors in HPV infection.

Psychological Factor	Description	Impact on HPV Infection	References
Stigma and shame	Negative emotions and social stigma related to sexually transmitted infections (STIs), particularly HPV.	Stigmatization can lead to avoidance of diagnosis and treatment, increasing the risk of HPV-related complications like cervical cancer. It also reduces open communication about prevention and treatment.	[75,76]
Fear and anxiety	Emotional responses such as fear of infection and anxiety about potential health risks related to HPV.	Fear and anxiety may lead individuals to delay or avoid necessary screenings and medical consultations, raising the risk of undiagnosed or untreated HPV-related diseases.	[76,77]
Self-esteem and body image	The perception of one’s body and self-worth, particularly in relation to the diagnosis of an STI like HPV.	Lower self-esteem due to the stigma of having an STI may result in poor engagement with medical care, hinder discussions about preventive behaviors, and contribute to further emotional distress.	[76,78]
Depression	Feelings of sadness, loss of interest in activities, and a lack of motivation to address health needs.	Depression can lead to neglect of health behaviors such as regular screenings, vaccination, and treatment adherence, resulting in worse health outcomes for individuals with HPV.	[76]
Perceived control	Belief in one’s ability to manage the course of an illness like HPV.	Individuals who feel they lack control over their health may be less likely to engage in preventive measures, follow treatment guidelines, or seek medical help, leading to worsened health outcomes.	[79,80]
Social support	Emotional, informational, and practical assistance provided by friends, family, and others.	Strong social support promotes better mental health, encourages positive health behaviors like vaccination and screenings, and leads to improved health outcomes in individuals with HPV.	[81]
Health literacy	Knowledge and understanding of health information, particularly regarding HPV transmission, prevention, and treatment.	Higher health literacy leads to better adherence to prevention methods (e.g., vaccination), more frequent screenings, and better management of HPV-related risks.	[76,82]
Coping mechanisms	Psychological strategies employed to deal with the stress or adversity caused by an HPV diagnosis.	Effective coping strategies (e.g., problem-solving, seeking social support) can result in healthier responses to HPV-related stress, adherence to treatment plans, and better overall health outcomes.	[83,84]
